# Late-Life Dietary Patterns and Subsequent Survival Among Adults Aged 90 Years or Older in the Sardinian Blue Zone

**DOI:** 10.3390/nu18183072

**Published:** 2026-09-20

**Authors:** Alessandra Errigo, Georgina Gómez, Laura Vindas-Meza, Giovanni Mario Pes

**Affiliations:** 1 Clinica Medica, Dipartimento di Medicina, Chirurgia e Farmacia, University of Sassari, Viale San Pietro 8, 07100 Sassari, Italy; alessandra.errig@tiscali.it; 2 Departamento de Bioquímica, Escuela de Medicina, Universidad de Costa Rica, Montes de Oca, San José 11501-2060, Costa Rica; georginagomezcr@gmail.com; 3 Asociación Península de Nicoya Zona Azul, San José 11301, Costa Rica; laura.vindasmeza@ucr.ac.cr

**Keywords:** Blue Zone, dietary patterns, longevity, Sardinia, elderly

## Abstract

**Background**: Dietary habits in long-lived populations are often interpreted as determinants of exceptional longevity, although most evidence is based on ecological or cross-sectional studies. In this study we examined whether diet measured after extreme old age was reached was associated with subsequent survival among Sardinian Blue Zone residents aged 90 years or older. **Methods**: The cohort included 200 participants aged 90–106 years who completed a qualitative food-frequency questionnaire covering 17 foods or food groups and were followed for up to seven years. A posteriori dietary patterns were derived using principal component analysis with Varimax rotation. Associations with all-cause mortality were assessed through Kaplan–Meier analysis and Cox models adjusted for age, sex, and comorbidity. **Results**: Four patterns were identified: dairy–traditional fat, red-meat, chicken–carbohydrate, and plant–starchy food. Greater conformity to the dairy–traditional-fat pattern was associated with lower mortality (HR per 1-SD increase, 0.839; 95% CI, 0.727–0.969, *p* = 0.017), although this pattern-level association did not meet the 5% false-discovery-rate (FDR) threshold (*q* = 0.066). The highest versus lowest tertile showed a similar association (HR, 0.682; 95% CI, 0.480–0.969). No other pattern was significantly associated with mortality. In secondary food-specific analyses, higher frequencies of milk, cheese, and chicken consumption were inversely associated with mortality, whereas wine consumption was positively associated with mortality; these four associations remained significant after FDR correction across the 17 foods examined. **Conclusions**: In adults aged 90 years or older, greater conformity to a dairy-based late-life dietary pattern was associated with longer subsequent survival, although the pattern-level association did not remain significant after FDR correction. These findings concern diet in extreme old age and should not be extrapolated to lifelong dietary factors influencing exceptional longevity.

## 1. Introduction

The influence of dietary habits on human lifespan has long been debated and recently attracted renewed scientific interest [1]. A substantial body of research has linked unhealthy dietary patterns to an increased risk of chronic non-communicable diseases [2] and higher mortality rates [3,4]. More recently, researchers have investigated exceptionally long-lived populations as potentially informative models for understanding the relationship between diet and longevity [5]. The traditional diets of these populations are often presumed to have contributed substantially to their exceptional survival, although these interpretations may oversimplify the complex, multifactorial nature of longevity [5]. In particular, dietary factors associated with attaining exceptional longevity should be distinguished from those associated with subsequent survival once extreme old age is reached.

Blue Zones are geographical areas characterized by unusually high levels of survival to advanced ages [6]. Among the most frequently cited examples are Okinawa in Japan [7], central-eastern Sardinia in Italy [6,8], the island of Ikaria in Greece [9] and the Nicoya Peninsula in Costa Rica [10]. Several descriptive studies have examined the dietary habits of these populations [11,12,13,14,15,16,17,18]. Although some broad dietary features recur across them, substantial geographical, historical, anthropological, and cultural differences make it difficult to identify a single dietary model consistently associated with the exceptional longevity of all these populations [5,14].

Much of the evidence linking traditional dietary habits to longevity is based on ecological or cross-sectional observations. Such studies cannot establish whether the dietary patterns observed in long-lived populations preceded or contributed to their exceptional survival and may not adequately account for demographic, socioeconomic, lifestyle, clinical, and other factors. Dietary habits may also change substantially over time [14,19], vary among subgroups within the same population [20], and produce different metabolic responses according to genetic background [21]. Consequently, the diet documented among a long-lived population at a point in time may not accurately represent that followed by the birth cohorts in which the longevity advantage originally emerged.

Prospective cohort studies provide a more suitable approach for evaluating temporal associations between dietary exposure and subsequent mortality [22]. By assessing diet at baseline and following participants over time, they allow subsequent survival to be compared among individuals with different dietary habits. Nevertheless, such studies remain uncommon in exceptionally long-lived populations. One of the few available investigations, conducted among Japanese centenarians, reported that a dietary pattern characterized by a preference for dairy products is associated with longer subsequent survival [23]. Comparable prospective evidence from scientifically identified Blue Zone populations remains very limited. Although observational follow-up cannot establish causality or reconstruct dietary exposure over the life course, it can directly evaluate whether diet measured in extreme old age is associated with subsequent survival. The present study was designed to address this knowledge gap.

The exceptional concentration of centenarians in central-eastern Sardinia was first reported in 1999 [8] and subsequently mapped and formally identified as a longevity Blue Zone in 2004 [6]. Since then, several studies have examined the potential contribution of nutrition to the exceptional longevity of the population in this region [5,14,15,24]. Within the broader framework of the Mediterranean dietary model, the traditional diet of the Sardinian Blue Zone has been described as centered predominantly on cereals and other plant-derived foods, with a substantial contribution from dairy products, moderate consumption of meat and eggs, and relatively limited fish consumption [14,24].

The health benefits of Mediterranean-style dietary patterns have been extensively documented, including lowering risks of cardiovascular disease, type 2 diabetes, certain cancers, and premature mortality [25,26,27,28,29]. Nevertheless, the Mediterranean diet is not a single, uniform model but encompasses dietary traditions that vary across geographical areas, historical periods, and population groups. In this context, evaluating overall dietary patterns rather than isolated nutrients or individual foods may provide a more realistic representation of habitual diet by capturing the combinations and correlations among foods consumed within a population.

Dietary patterns may be evaluated using either a priori or a posteriori approaches [30,31,32]. A priori methods quantify adherence to dietary models defined in advance based on nutritional knowledge, dietary guidelines, or established evidence on diet–disease relationships. Examples include the Mediterranean Diet Score, the Healthy Eating Index, and the Dietary Approaches to Stop Hypertension score [33,34]. By contrast, a posteriori methods derive dietary patterns empirically from the dietary data collected in the study population, without classifying foods in advance as beneficial or harmful. Commonly used techniques include principal component analysis (PCA), exploratory factor analysis, and cluster analysis, although these methods address different analytical objectives. PCA reduces a set of correlated food variables to a smaller number of linear components that capture the principal sources of variation in reported dietary intake. Each participant receives a component score indicating the degree to which their dietary profile conforms to each identified pattern, and components are interpreted and labeled according to the foods with the largest absolute loadings [30,31,32].

Against this background, the present study investigated the association between empirically derived, a posteriori dietary patterns and subsequent survival among Sardinian Blue Zone residents enrolled at age 90 years or older and followed for up to seven years.

## 2. Materials and Methods

### 2.1. Study Design

This prospective cohort study included adults aged 90 years or older residing in the Sardinian Blue Zone of central-eastern Sardinia, Italy. Participants were enrolled during two baseline recruitment waves conducted in 2018 [35] and 2023 [36] and followed from the date of their individual baseline assessment. The study protocol was approved by the Institutional Ethics Committee of the Azienda Ospedaliero-Universitaria di Sassari (Prot. No. 136/CE, 9 February 2012). Written informed consent was obtained from participants who were able to provide it. For individuals with severe cognitive impairment who lacked such capacity, consent was instead obtained from a legally authorized representative.

### 2.2. Study Participants

Details of the study population and recruitment procedures have been reported previously [35]. Participants were eligible for inclusion in the study if they were aged 90 years or older at recruitment, were of Sardinian ancestry, and had all four grandparents born within the Sardinian Blue Zone. Potentially eligible individuals were identified through the population registers of six municipalities located within the Blue Zone area. During the first recruitment wave in 2018, 331 eligible residents aged 90 years or older were invited to participate in the study, of whom 150 were enrolled (61 men and 89 women), corresponding to a participation rate of 45.3%. In 2023, a second recruitment wave based on convenience sampling was conducted to increase the cohort to 200 participants; 50 additional adults aged 90 years or older were enrolled (39 men and 11 women) [36]. The same eligibility criteria were applied in both waves. As the 2023 wave did not involve systematic enumeration and invitation of all eligible residents, a denominator was not available and a conventional participation rate could not be calculated. The final study cohort therefore comprised 200 participants, each of whom entered follow-up from the date of their baseline assessment.

### 2.3. Data Collection

At recruitment, the participants underwent a face-to-face interview at their place of residence, conducted by trained personnel using standardized procedures [35]. Dietary data and demographic and clinical variables relevant to the present analyses were collected. A broader multidimensional geriatric assessment, including cognitive and functional measures, was also performed as described previously [35].

Vital status during follow-up was ascertained through official municipal population registers. For the participants who had died, the exact date of death was obtained from the relevant municipal registry. Follow-up time was calculated from the date of the baseline assessment to the date of death or, for the participants who were still alive, to the date of the final vital-status verification.

### 2.4. Dietary Assessment

Dietary habits at baseline were assessed using a short qualitative food-frequency questionnaire (FFQ) previously employed in nutritional surveys conducted in the Sardinian Blue Zone [14]. All participants were able to consume food orally at baseline, and none were receiving enteral nutrition. Information on medically prescribed or disease-specific diets was not collected systematically. The participants reported their usual consumption frequency for 17 foods or food groups: beef/pork meat, sheep/goat meat, chicken, fish, pulses, leafy green vegetables, fruit, bread, pasta, potatoes, olive oil, lard, traditional homemade sweets or biscuits, cheese (including traditional soft sour cheese), milk, coffee, and wine. Consumption was recorded using five predefined frequency categories: never or rarely, 2–3 times per month, 1–2 times per week, 3–5 times per week, and daily. Since the FFQ was qualitative, portion sizes were not recorded and total energy intake could not be estimated. Consequently, energy intake was not available for energy adjustment of the food-frequency variables before PCA or as a covariate in the survival models. Total energy intake was nevertheless considered a potential source of residual confounding in the diet–mortality associations.

### 2.5. Covariates

Age at recruitment and sex were identified a priori as potential confounders. Comorbidity burden was assessed at baseline using a simplified measure derived from the Cumulative Illness Rating Scale (CIRS) [37]. Fourteen medical categories were considered, and for each category the presence or absence of disease was recorded dichotomously, without grading disease severity. The resulting CIRS-derived comorbidity count corresponded to the number of medical categories affected by at least one condition, with higher values indicating greater comorbidity burden Age, sex, and CIRS-derived comorbidity count were included as covariates in the multivariable Cox proportional hazards models. Cognitive status was assessed using the Mini-Mental State Examination (MMSE) [38], functional autonomy using the six-item Basic Activities of Daily Living (BADL) scale [39], and anthropometric status using calf circumference [40]. Higher MMSE and BADL scores indicated better cognitive performance and greater functional independence, respectively. These measures were included, together with age, sex, and the CIRS-derived comorbidity count, in an additional sensitivity analysis to assess whether cognitive status, functional autonomy, and anthropometric status materially influenced the associations between dietary-pattern scores and mortality.

Physical activity was assessed from the reported frequency of walking outside the home and dichotomized as walking outside the home at least three times per week versus less than three times per week. Because this measure could reflect functional reserve as well as habitual activity in very old individuals, it was not included in the primary model but was examined as an additional sensitivity covariate.

### 2.6. Statistical Analysis

Statistical analyses were performed using R software, version 4.5.1 (R Foundation for Statistical Computing, Vienna, Austria). Continuous variables were summarized as mean and standard deviation (SD) or median and interquartile range (IQR), as appropriate, whereas categorical variables were summarized as counts and percentages. Baseline characteristics were also summarized separately by recruitment wave. Between-wave differences were quantified descriptively using absolute standardized mean differences (SMDs); formal hypothesis testing was not used because the 2023 wave was based on convenience sampling rather than an independently sampled comparison group.

#### 2.6.1. Derivation of Dietary Patterns

A posteriori dietary patterns were derived from baseline food-frequency data using a PCA. For the analysis, the five consumption-frequency categories were converted into estimated weekly frequencies as follows: never or rarely, 0 times per week; 2–3 times per month, 0.625 times per week; 1–2 times per week, 1.5 times per week; 3–5 times per week, 4 times per week; and daily, 7 times per week. The 17 food-frequency variables were standardized as Z-scores, and a PCA was performed on their correlation matrix. The PCA was therefore performed on standardized consumption-frequency variables rather than energy-adjusted dietary intakes. Accordingly, the resulting components represent patterns of covariation in reported food-consumption frequency and should not be interpreted as an energy-adjusted measure of dietary composition. Methodological studies have shown that dietary patterns derived using a PCA from unadjusted and energy-adjusted FFQ variables are often broadly similar, although some differences in component loadings may occur [34,41].

The suitability of the dietary data for the PCA was assessed using the Kaiser–Meyer–Olkin measure of sampling adequacy and Bartlett’s test of sphericity [42,43]. A Kaiser–Meyer–Olkin value greater than 0.50 and a statistically significant Bartlett’s test were considered evidence supporting the adequacy of the correlation structure. Horn’s parallel analysis was performed using the *fa.parallel()* function in the *psych* package (fa = “pc”, 5000 iterations, 95th percentile of simulated eigenvalues; random seed = 2026). The retained components were estimated using the *principal()* function in the *psych* package with orthogonal Varimax rotation and regression-based component scores.

Food items with an absolute rotated component loading of at least 0.40 were considered relevant for interpretation. Components were labeled according to the foods with the largest absolute positive and negative loadings. Since the signs of PCA components are arbitrary, their orientation was chosen so that the principal foods defining each pattern had positive loadings. Participant-specific component scores were calculated using the regression method and subsequently standardized to a mean of 0 and an SD of 1. Higher scores therefore indicated greater conformity to the dietary profile represented by the positive loadings and lower consumption of foods with negative loadings.

#### 2.6.2. Survival Analysis

Follow-up time was calculated for each participant from the date of the baseline assessment to the date of death. The outcome was death from any cause. All participants had died by the end of follow-up; therefore, no observations were censored.

In the primary pattern-based analyses, component scores were examined as continuous variables, with hazard ratios (HRs) expressed per 1-SD increase. Cox proportional hazards regression models were used to estimate HRs and 95% confidence intervals (CIs) for associations between dietary-pattern scores and all-cause mortality. Model 1 was unadjusted, Model 2 was adjusted for age at recruitment and sex, and Model 3 was adjusted for comorbidity burden, as measured by the CIRS-derived comorbidity count. As additional sensitivity analyses, the continuous dietary-pattern models were further adjusted for MMSE score, BADL score, and calf circumference; for physical activity, defined as walking outside the home at least three times per week versus less frequently; and for recruitment wave. A model including both physical activity and recruitment wave was also fitted. To evaluate the robustness of the findings for the two-wave recruitment design, the primary continuous-score models were repeated among the 150 participants recruited in 2018, and potential heterogeneity between recruitment waves was explored by including a dietary-pattern score × recruitment-wave interaction term. Benjamini–Hochberg false-discovery-rate correction was applied across the four dietary-pattern tests separately for each sensitivity analysis and across the four pattern-by-wave interaction tests. In a supplementary mutually adjusted model, all four continuous pattern scores were entered simultaneously together with age, sex, and CIRS-derived comorbidity count. Cox proportional hazards models were fitted using the *coxph()* function in the *survival* package, with tied event times handled by the Efron approximation.

For descriptive and categorical analyses, component scores were divided into tertiles based on their empirical distributions. Tied values at a cutoff were retained within the same category, and tertile sizes could thus be unequal. Kaplan–Meier curves were constructed for each pattern and compared using global log-rank tests. Median survival times and survival probabilities at three and five years were estimated by tertile. Pairwise log-rank comparisons were adjusted using the Benjamini–Hochberg procedure. Fully adjusted Cox models compared the second and third tertiles with the first tertile as the reference category. The overall contribution of each tertile variable was evaluated using a likelihood-ratio test, and the four global tertile tests were additionally corrected for FDR.

The proportional-hazards assumption was assessed using tests based on scaled Schoenfeld residuals. Where evidence of non-proportionality was detected, Cox-model HRs were interpreted as average associations over the follow-up period rather than as constant effects. To investigate possible reverse causation from preterminal dietary change, sensitivity analyses repeated the fully adjusted continuous-score models after excluding participants who died within the first six months and within the first year of follow-up. FDR correction was applied across the four dietary-pattern tests separately at each exclusion threshold.

Associations between individual foods and mortality were examined in secondary exploratory analyses. Each of the 17 food-frequency variables was entered separately into a Cox model adjusted for age, sex, and CIRS-derived comorbidity count; foods were not mutually adjusted for one another. The primary food-specific HRs were expressed per 1-SD increase in estimated weekly consumption frequency. Estimates per one additional occasion of consumption per week were also calculated for descriptive purposes. The 17 food-specific *p*-values were corrected using the Benjamini–Hochberg FDR procedure [44]. All tests were two-sided. Nominal *p*-values and FDR-adjusted q-values were calculated, with values below 0.05 considered statistically significant.

## 3. Results

### 3.1. Characteristics of the Study Population

The study cohort comprised 200 adults aged 90 years or older, including 100 men and 100 women (Table 1). The mean age at recruitment was 93.6 ± 4.4 years, and the median age was 91 years (IQR, 90–97; range, 90–106 years). Women were older at recruitment than men, with mean ages of 94.5 ± 4.6 and 92.8 ± 4.1 years, respectively.

Baseline characteristics in both recruitment waves are shown separately in Supplementary Table S1. Participants recruited in 2023 were older than those recruited in 2018 (97.48 ± 5.09 vs. 92.33 ± 3.33 years) and included higher proportions of men (78.0% vs. 40.7%) and centenarians (50.0% vs. 6.7%), consistent with the different sampling approach used in the second wave. In contrast, CIRS-derived comorbidity burden, BADL score, and calf circumference were broadly similar between waves (absolute SMDs, 0.18, 0.10, and 0.09, respectively), whereas MMSE score was somewhat lower among participants recruited in 2023 (17.78 ± 8.04 vs. 19.98 ± 6.02; SMD, 0.31).

All participants had died by the final ascertainment of vital status; therefore, no observations were censored. The mean follow-up time was 3.27 ± 1.81 years, and the median follow-up was 3.30 years (IQR, 1.68–4.50; range, 0.10–7.00 years). The cohort contributed 653.4 person-years of observation, corresponding to a crude mortality rate of 30.61 deaths per 100 person-years. Mean follow-up was 3.69 ± 1.81 years in men and 2.85 ± 1.72 years in women. The mean CIRS-derived comorbidity count was 1.80 ± 1.14, with a median of 2 (IQR, 1–2).

All 200 participants consumed food orally, and none received enteral nutrition at baseline.

### 3.2. Identification of Dietary Patterns

A PCA was performed using the 17 baseline food-frequency variables for all 200 participants. The overall Kaiser–Meyer–Olkin measure of sampling adequacy was 0.660, and Bartlett’s test of sphericity was statistically significant, χ^2^ = 789.51, 136 degrees of freedom, *p* < 0.001, supporting the suitability of the correlation matrix for PCA.

Six components had eigenvalues greater than 1.0; however, inspection of the scree plot, Horn’s parallel analysis, and the interpretability of the resulting solution supported the retention of four components (Supplementary Figure S1). Together, the four retained components accounted for 49.6% of the total variance in the standardized food-frequency variables. After Varimax rotation, PC1 accounted for 14.99% of the variance, PC2 for 12.53%, PC3 for 11.92%, and PC4 for 10.15%.

PC1 was characterized by positive loadings for cheese, including traditional soft sour cheese (0.787), milk (0.786), and lard (0.436), and inverse loadings for olive oil (−0.673) and coffee (−0.465). It was therefore labeled the dairy–traditional-fat pattern. PC2, labeled the red-meat pattern, was characterized by positive loadings for goat meat (0.686), beef (0.672), and leafy green vegetables (0.449), and inverse loadings for lard (−0.487) and sweets (−0.462). PC3, labeled the chicken–carbohydrate pattern, was characterized by positive loadings for chicken (0.780), bread (0.463), pasta (0.481), and sweets (0.484), and an inverse loading for fish (−0.466). PC4 was characterized by positive loadings for potatoes (0.679), fruit (0.554), and pulses (0.467), and a strong inverse loading for wine (−0.635). It was labeled the plant–starchy-food pattern (Table 2).

Participant-specific component scores were calculated and standardized to a mean of 0 and an SD of 1. Higher scores indicated greater conformity to the dietary profile represented by the positive loadings and lower consumption of foods with negative loadings. For the categorical and Kaplan–Meier analyses, component scores were additionally divided into tertiles.

Loading plots illustrating the positions of the food-frequency variables in the planes defined by the rotated components are shown in Supplementary Figure S2.

### 3.3. Survival Analysis

**Figure 1:**
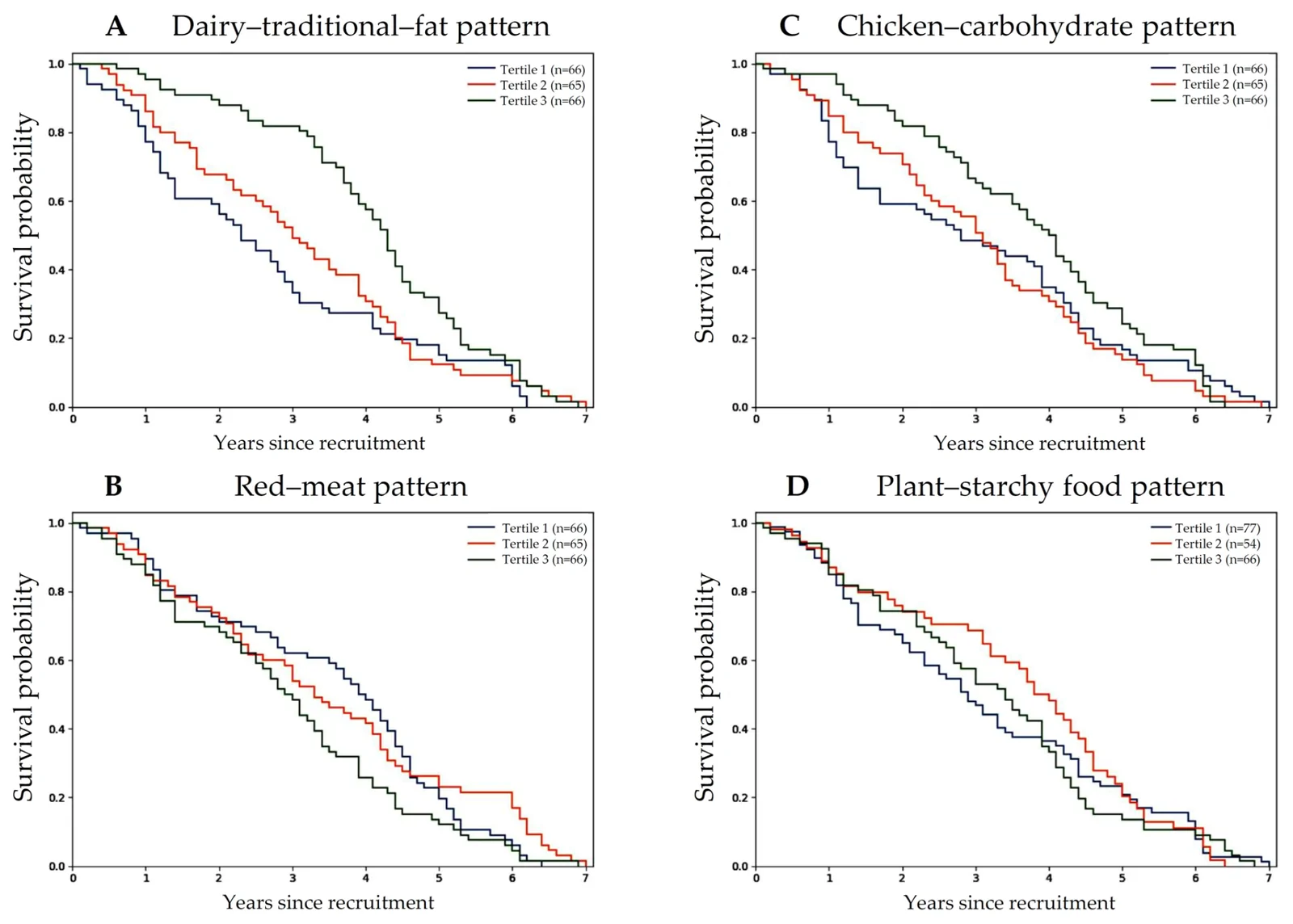
Kaplan–Meier survival curves according to tertiles of dietary-pattern scores. Survival from formal recruitment at age 90 years or older is shown according to tertiles of the (**A**) dairy–traditional fat, (**B**) red-meat, (**C**) chicken–carbohydrate, and (**D**) plant–starchy–food pattern scores. Tertile 1 indicates the lowest conformity and tertile 3 the highest conformity to the corresponding dietary pattern. Differences among curves were evaluated using the log-rank test.

Kaplan–Meier survival curves according to tertiles of the four dietary-pattern scores are shown in Figure 1. Survival distributions differed significantly across tertiles of the dairy–traditional-fat pattern (Figure 1A; log-rank χ^2^ = 12.17, *p* = 0.0023; FDR-adjusted *q* = 0.0091). Estimated median survival increased from 2.3 years in the lowest tertile to 2.85 years in the middle tertile and 4.3 years in the highest tertile. The corresponding probabilities of surviving for at least three years after recruitment were 35.8%, 45.5%, and 80.6%, respectively. Benjamini–Hochberg-adjusted pairwise comparisons showed longer survival in the highest tertile than in both the lowest (*q* = 0.001) and middle tertiles (*q* = 0.013), whereas the lowest and middle tertiles did not differ significantly (*q* = 0.351; see Supplementary Table S2).

No statistically significant differences were observed in survival distributions across tertiles of the red-meat pattern (Figure 1B; log-rank χ^2^ = 2.47, *p* = 0.291), the chicken–carbohydrate pattern (Figure 1C; χ^2^ = 3.01, *p* = 0.222), or the plant–starchy-food pattern (Figure 1D; χ^2^ = 0.48, *p* = 0.787). For the red-meat pattern, estimated median survival declined from 3.9 years in the lowest tertile to 3.2 years in the middle tertile and 3.0 years in the highest tertile; however, the global difference was not statistically significant.

Cox regression results are presented in Table 3. In the unadjusted analysis, each 1-SD increase in the dairy–traditional-fat pattern score was linked to a lower hazard of death (HR, 0.785; 95% CI, 0.684–0.901; *p* = 0.0006). The association remained nominally significant after adjustment for age and sex (HR, 0.837; 95% CI, 0.725–0.966; *p* = 0.015) and additional adjustment for CIRS-derived comorbidity count (HR, 0.839; 95% CI, 0.727–0.969; *p* = 0.017). Thus, in the fully adjusted model, each 1-SD higher score corresponded to an estimated 16.1% lower average hazard of death over follow-up. However, the association did not meet the 5% FDR threshold after correction across the four dietary-pattern tests (*q* = 0.066).

In the categorical analysis, the participants in the highest tertile of the dairy–traditional-fat pattern had a 31.8% lower adjusted average hazard of death than those in the lowest tertile (HR, 0.682; 95% CI, 0.480–0.969; *p* = 0.033); moreover, the middle tertile did not differ from the lowest tertile (HR, 0.996; 95% CI, 0.698–1.420; *p* = 0.980). The global likelihood-ratio test for the tertile variable was nominally significant (*p* = 0.041), although it did not remain significant after correction across the four dietary-pattern tests (*q* = 0.165). None of the other dietary patterns was significantly associated with mortality in the categorical analyses.

In the mutually adjusted model including all four continuous dietary-pattern scores together with age, sex, and CIRS-derived comorbidity count, a 1-SD higher dairy–traditional-fat pattern score remained associated with a lower average hazard of death (HR, 0.832; 95% CI, 0.718–0.964; *p* = 0.014). The red-meat (HR, 1.052; 95% CI, 0.909–1.217; *p* = 0.495), chicken–carbohydrate (HR, 0.962; 95% CI, 0.838–1.103; *p* = 0.575), and plant–starchy-food patterns (HR, 0.987; 95% CI, 0.852–1.143; *p* = 0.858) were not significantly associated with mortality in this model (Supplementary Table S3). As part of an additional sensitivity analysis, the continuous dietary-pattern models were further adjusted for cognitive status, functional autonomy, and calf circumference. The association for the dairy–traditional–fat pattern was only modestly attenuated (HR per 1-SD increase, 0.852; 95% CI, 0.736–0.986; *p* = 0.032; FDR *q* = 0.127), compared with HR 0.839 in the primary model. The other three dietary patterns remained unassociated with mortality (Supplementary Table S4). Sensitivity analyses excluding early deaths yielded broadly similar effect estimates. After the eight participants who died within the first six months of follow-up were excluded (n = 192), the dairy–traditional–fat pattern remained inversely associated with mortality (HR per 1-SD increase, 0.852; 95% CI, 0.736–0.985; *p* = 0.031; FDR *q* = 0.124). After the 28 participants who died within the first year were excluded (n = 172), the association was modestly attenuated and no longer reached conventional statistical significance (HR, 0.865; 95% CI, 0.742–1.008; *p* = 0.063; *q* = 0.252). The other three dietary patterns remained unassociated with mortality in both sensitivity analyses (Supplementary Table S5).

Additional sensitivity analyses addressing physical activity and recruitment wave yielded stable estimates (Supplementary Table S6). Adjustment for recruitment wave did not materially alter the association between the dairy–traditional–fat pattern and mortality (HR per 1-SD increase, 0.838; 95% CI, 0.726–0.967; *p* = 0.016). Further adjustment for physical activity produced a similar estimate (HR, 0.832; 95% CI, 0.720–0.961; *p* = 0.012), which essentially remained unchanged when recruitment wave and physical activity were included simultaneously (HR, 0.832; 95% CI, 0.720–0.961; *p* = 0.012; FDR *q* = 0.049). When the analysis was restricted to the 150 participants recruited in 2018, the association remained in the same direction but was attenuated and less precise (HR, 0.882; 95% CI, 0.747–1.043; *p* = 0.142). No evidence of heterogeneity in the dairy–traditional–fat association was found between recruitment waves (interaction *p* = 0.252). The other dietary-pattern associations remained largely unchanged in these sensitivity analyses.

### 3.4. Individual Food Items and Survival

**Figure 2:**
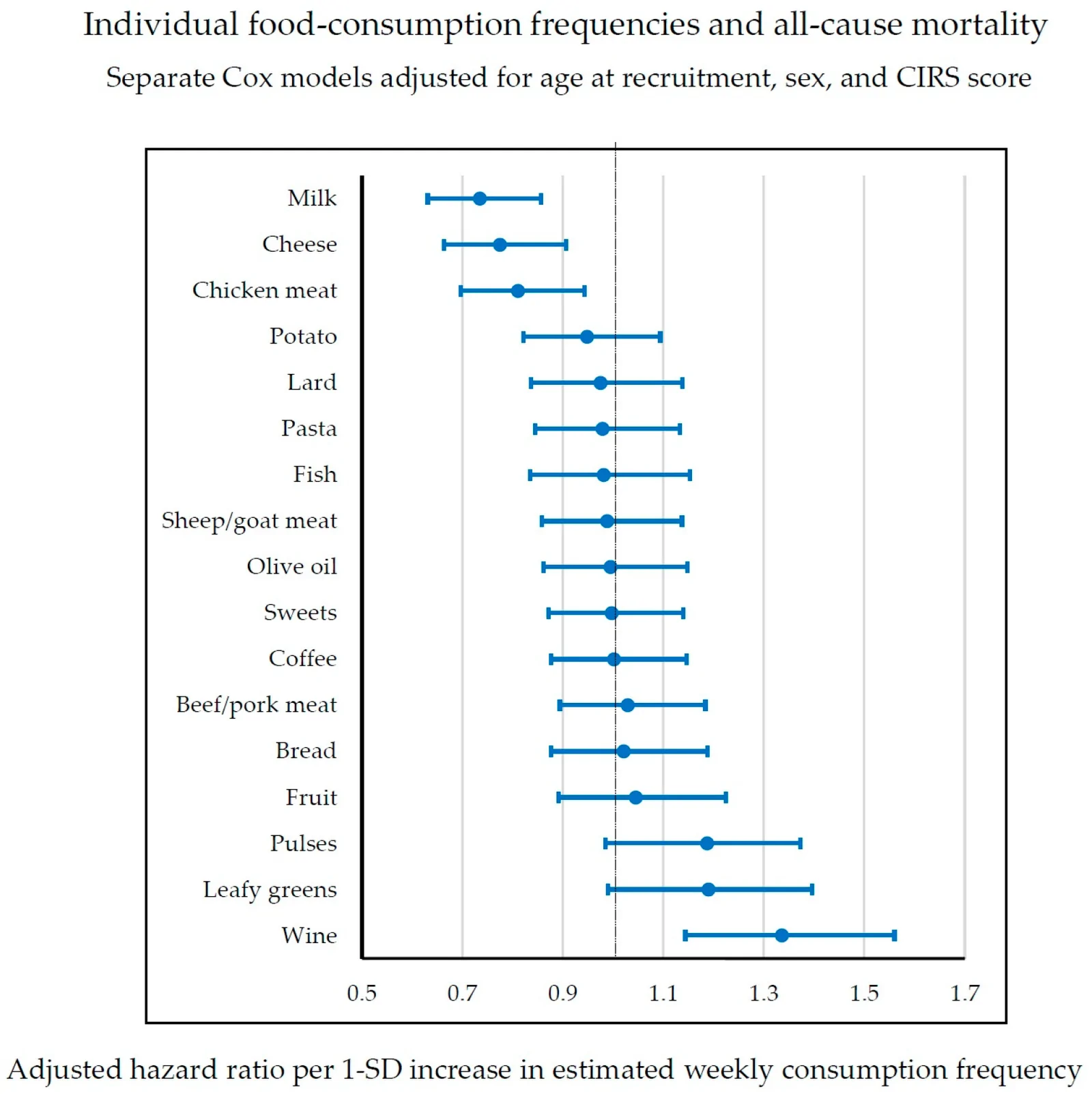
Associations between individual food-consumption frequencies and all-cause mortality. Hazard ratios and 95% confidence intervals were estimated using separate Cox proportional hazards models adjusted for age at recruitment, sex, and CIRS-derived comorbidity count and are expressed per 1-SD increase in estimated weekly consumption frequency. The vertical reference line indicates an HR of 1.00. Milk, cheese, chicken, and wine remained statistically significant after Benjamini–Hochberg false-discovery-rate correction across the 17 food-specific analyses. CIRS, Cumulative Illness Rating Scale; HR, hazard ratio; SD, standard deviation.

In secondary exploratory analyses, each of the 17 food-frequency variables was examined separately in a Cox proportional hazards model adjusted for age, sex, and CIRS-derived comorbidity count. Hazard ratios were expressed per 1-SD increase in estimated weekly consumption frequency. After Benjamini–Hochberg correction across the 17 comparisons, higher consumption frequencies of milk (HR, 0.735; 95% CI, 0.631–0.857; *q* = 0.0014), cheese (HR, 0.775; 95% CI, 0.663–0.906; *q* = 0.0077), and chicken (HR, 0.811; 95% CI, 0.697–0.943; *q* = 0.0275) were associated with a lower hazard of death, whereas wine consumption was associated with a higher hazard (HR, 1.336; 95% CI, 1.144–1.561; *q* = 0.0022). No other individual food remained statistically significant after FDR correction (Figure 2 and Supplementary Table S7).

## 4. Discussion

This prospective study of residents of the Sardinian Blue Zone recruited at age 90 years or older found that greater conformity to a dairy–traditional-fat dietary pattern was associated with a lower average hazard of death during follow-up after adjustment for age, sex, and comorbidity. A similar association was observed when the highest and lowest tertiles of the pattern score were compared. The food-specific analyses were broadly concordant: higher consumption frequencies of milk and cheese showed the strongest inverse associations with mortality, chicken showed a weaker inverse association, and higher wine-consumption frequency was associated with a higher hazard of death; all four associations remained significant after FDR correction. These findings concern diet measured in extreme old age and subsequent survival; they do not identify the dietary determinants of attaining exceptional longevity. Taken together, they suggest that the consumption of nutrient-dense foods, particularly dairy products, may be a marker of greater nutritional resilience in very late life.

Our previous cross-sectional study in the Sardinian Blue Zone found that cheese consumption was associated with better self-rated health, cognitive function, autonomy in activities of daily living, and calf circumference, whereas chicken consumption was associated with a lower disease burden and more favorable affective and physical health indicators [14]. The present prospective findings complement these observations by showing that the same foods are also associated with subsequent survival. Together, the results suggest that foods associated with preserved nutritional and functional reserve in extreme old age may also be related to longer subsequent survival. Importantly, however, dietary factors associated with survival after recruitment at age 90 years or older need not be the same as those that contributed to reaching such an exceptional age.

Several nutritional pathways could plausibly underlie the observed association with dairy consumption. In very old adults, milk and cheese provide energy, high-quality protein, calcium, and other micronutrients that may help maintain muscle mass, functional reserve, and resilience to acute illness. Evidence from older populations suggests that dairy food consumption may be associated with a lower risk of frailty and sarcopenia, although findings remain heterogeneous and direct evidence of a survival benefit is limited [45]. The present results are also consistent with a prospective study of Japanese centenarians in which a dietary pattern characterized by a preference for dairy products was associated with longer subsequent survival [23]. The associations observed for milk and cheese may thus partly reflect the value of nutrient-dense foods in supporting nutritional status and physiological reserve in extreme old age.

In the Sardinian Blue Zone, the broad cheese category likely included *casu axedu* (also known as *casu ajedu*), a traditional acidic fermented product made from sheep’s or goat’s milk and historically common in pastoral households [24,46]. In an earlier survey of oldest-old residents from the same area, 77.5% of women and 83.6% of men reported consuming *casu axedu* between one and five times per week, confirming that it was a customary rather than occasional food [13]. Experimental supplementation in rats altered gut microbial composition and function and reduced selected microbial virulence and host inflammatory markers [47]. Although these findings provide some biological plausibility, they do not demonstrate a survival benefit in humans. Moreover, because the present FFQ did not distinguish among cheese varieties, the association observed for cheese cannot be attributed specifically to *casu axedu*.

The inverse association with chicken consumption was weaker than that observed for dairy foods and was not reproduced by the broader chicken–carbohydrate pattern; it should therefore be interpreted cautiously. Chicken nevertheless provides high-quality protein, which may be particularly relevant in extreme old age, when anabolic resistance and vulnerability to catabolic stress increase protein requirements [48]. Previous research in the Sardinian Blue Zone also associated greater chicken consumption with better performance in activities of daily living [14], while prospective evidence suggests that poultry is generally not associated with increased mortality and may be a preferable alternative to red or processed meat [49]. Chicken intake may also be associated with better appetite, functional capacity, food availability, or household support. The absence of an association with fish may partly reflect its traditionally low and relatively uniform consumption in this predominantly agro-pastoral population [14].

An alternative interpretation is that late-life food consumption partly reflects underlying health and functional status. In very old adults, frailty, loss of appetite, cognitive impairment, reduced ability to shop or prepare food, dysphagia, and increasing dependence in eating may substantially modify both the amount and type of foods consumed [50]. These conditions are themselves associated with mortality and may therefore generate or strengthen apparent associations between specific foods and subsequent survival. This possibility is particularly relevant for the food-specific findings for dairy products and chicken. In the Sardinian pastoral setting, cheese has historically been an inexpensive and readily available component of the habitual diet, which makes socioeconomic access a less obvious explanation for differences in consumption; nevertheless, declining health or feeding autonomy can still reduce its consumption in late life. In the present study additional adjustment for MMSE, BADL, and calf circumference only modestly attenuated the dairy-pattern estimate, but these measures cannot fully capture multidimensional frailty, appetite, swallowing ability, or dependence in food preparation and feeding. Residual confounding and reverse causation thus remain possible.

The absence of an association with the plant–starchy-food pattern should not be interpreted as evidence against the benefits of plant-rich diets. This empirically derived component, characterized mainly by potatoes, fruit, and pulses, was not equivalent to a comprehensive Mediterranean or plant-based dietary score. Moreover, the participants were highly selected survivors who had already reached at least 90 years of age; dietary factors that reduced chronic disease risk earlier in life may thus have been less strongly related to their subsequent survival. Restricted variation in food consumption, the limited resolution of the qualitative FFQ, and illness- or frailty-related dietary changes may also have attenuated associations. These findings concern the prognostic relevance of diet in extreme old age and do not contradict prospective evidence linking Mediterranean dietary patterns to lower mortality in younger older adults [51,52].

The absence of favorable associations with pulses and leafy green vegetables should likewise be interpreted cautiously. Neither food-specific association remained significant after FDR correction, and the present study did not assess gastrointestinal tolerance, nutrient bioavailability, or other factors that might influence consumption or nutritional effects in extreme old age. These findings should therefore not be interpreted as evidence of adverse effects of these foods.

Olive oil consumption was not associated with subsequent survival in the present cohort. This null finding is not necessarily inconsistent with studies linking Mediterranean dietary patterns or greater olive-oil intake to lower mortality, nor with our previous observation that increasing olive oil consumption during the Sardinian nutrition transition was associated with favorable concurrent health indicators [14]. The earlier study examined retrospective dietary change and cross-sectional health, whereas the present analysis related consumption frequency measured after recruitment at age 90 years or older to subsequent mortality. Moreover, the FFQ recorded neither the amount nor the type of olive oil consumed, although associations may vary according to dose and whether the oil is virgin or refined [53]. In extreme old age, the preservation of nutritional status, muscle function, and physiological reserve may become more immediately relevant to subsequent survival than dietary effects operating mainly through long-term cardiometabolic pathways. A late-life cholesterol paradox was also observed locally [54]. Although this interpretation remains hypothetical, it is consistent with evidence linking malnutrition and sarcopenia to functional decline and mortality in older adults [55].

Higher wine consumption frequency was associated with a greater hazard of death after adjustment for age, sex, and comorbidity, and the association remained significant after FDR correction. This finding is noteworthy because red wine, particularly Cannonau, is frequently portrayed as a beneficial component of the traditional Sardinian diet due to its polyphenol content [11,56], although earlier Sardinian ecological findings also suggested a weak adverse association [15]. Reverse causation deserves consideration in this exceptionally old population. Declining health, increasing frailty, or medication use may lead some individuals to reduce or discontinue alcohol consumption, whereas healthier nonagenarians may be more likely to maintain customary or socially embedded drinking habits. Such a “sick-quitter” mechanism, however, generally tends to favor rather than disadvantage wine consumers and therefore does not readily account for the higher mortality observed with increasing wine consumption frequency. Conversely, a detrimental contribution of ethanol remains biologically plausible at very advanced ages, when reduced physiological reserve, multimorbidity, polypharmacy, nutritional vulnerability, and susceptibility to falls may increase sensitivity to its adverse effects. More generally growing evidence indicates that low-volume alcohol consumption should not be assumed to confer a survival advantage. Among adults aged 60 years or older, even low-risk drinking has been associated with higher mortality in individuals with health-related or socioeconomic vulnerability [57], while meta-analyses addressing former-drinker misclassification and other reference-group biases have found no reduction in all-cause mortality among low-volume drinkers compared with lifetime abstainers [58]. Risk-modeling studies likewise indicate no net protective effect at low levels of consumption, and the World Health Organization Europe states that no level of alcohol intake can currently be considered safe for health [59,60]. Thus, the presence of potentially beneficial polyphenols in wine does not negate the health risks attributable to ethanol. Nevertheless, because the FFQ did not quantify wine volume or ethanol dose, distinguish former drinkers from lifetime abstainers, or capture drinking context and lifetime changes in alcohol use, the present observational data cannot establish that ethanol itself caused the excess mortality. They do, however, provide no support for considering wine a health-promoting component of the late-life Sardinian diet.

This study has several strengths. It prospectively examined an exceptionally old population rarely represented in nutritional research, achieved complete ascertainment of vital status, and combined pattern-based with food-specific analyses that produced broadly concordant findings for dairy foods. Interpretation was further supported by the well-characterized cultural context of the Sardinian Blue Zone and previous local observations linking cheese and chicken consumption with favourable health and functional indicators [14].

Several limitations should nonetheless be considered. Diet was assessed only at baseline, and reverse causation cannot be excluded: lower consumption of milk, cheese, chicken, or other foods may have reflected anorexia, poor dentition, dysphagia, frailty, illness, or symptom-driven avoidance rather than an effect of diet on survival. Reverse causation remains an important consideration because declining health, reduced appetite, dysphagia, functional impairment, or other manifestations of terminal decline may alter dietary intake before death. The inverse association with the dairy–traditional-fat pattern was essentially preserved after excluding deaths within the first six months, which raises an argument against very short-term terminal decline as the sole explanation. However, the estimate was modestly attenuated and less precise after exclusion of deaths within the first year, so illness-related dietary change before recruitment cannot be excluded.

The attenuation of the dairy-pattern association after excluding deaths occurring within the first year is consistent with some contribution of illness-related dietary change or residual confounding, although the effect estimate remained in the same direction. In addition, the participants had already survived to at least age 90, creating a highly selected population in which dietary influences operating earlier in life may no longer have been detectable.

Residual confounding may nevertheless have remained from aspects of physical and cognitive function not fully captured by the available measures, as well as from socioeconomic circumstances, living arrangements, smoking history, and caregiver support. Reassuringly, the dairy–traditional-fat association changed little in sensitivity models additionally accounting for physical activity, cognitive status, functional autonomy, anthropometric status, and recruitment wave. The qualitative FFQ recorded consumption frequency but not portion size, preventing estimation of total energy intake. Although a PCA based on food-frequency variables does not inherently require energy adjustment and previous methodological work has found broadly similar patterns using energy-adjusted and unadjusted FFQ data [41], the present component scores may partly reflect differences in overall food consumption rather than dietary composition alone. Moreover, residual confounding by total energy intake could not be evaluated in the survival models.

Information on medically prescribed or disease-specific diets was not systematically collected. This may be relevant because chronic conditions capable of prompting dietary modification were present in the cohort; for example, a previous analysis of the same study population reported diabetes in approximately 12% of participants [36]. We therefore cannot exclude that physician advice, self-imposed dietary restrictions, or other disease-related dietary modifications influenced the reported consumption of particular foods and, consequently, the empirically derived dietary patterns. Adjustment for overall comorbidity burden may have reduced, but cannot eliminate, this potential source of confounding.

Although cognitive status, functional autonomy, and comorbidity burden were characterized at baseline, these measures were not repeatedly assessed during follow-up. The present study thus evaluates subsequent survival rather than healthspan and cannot determine whether longer survival was accompanied by preservation of physical function, autonomy, cognition, or quality of life.

An additional limitation is survivor selection inherent in the recruitment of individuals who had already reached at least 90 years of age. By definition, the cohort represents an exceptionally selected subset of the source population. Individuals who were more susceptible to adverse dietary, metabolic, or behavioral exposures may have died before becoming eligible for recruitment, whereas those included in the study may possess biological, behavioral, or environmental characteristics associated with exceptional survival. Conditioning the analysis on survival to such advanced ages may therefore attenuate, obscure, or even modify associations with dietary factors that exert stronger effects earlier in the life course. Accordingly, the present findings should be interpreted specifically as associations between dietary habits measured in extreme old age and subsequent survival after recruitment, and not as evidence regarding the effects of these dietary patterns across adulthood or their role in determining the attainment of exceptional longevity.

## 5. Conclusions

Among Sardinian Blue Zone residents recruited at age 90 years or older, greater conformity to a dairy–traditional-fat dietary pattern was associated with a lower average hazard of death after adjustment for age, sex, and comorbidity. Higher consumption frequencies of milk, cheese, and chicken were associated with lower mortality, whereas higher wine-consumption frequency was associated with higher mortality. These findings concern diet in extreme old age and subsequent survival, not the lifelong dietary determinants of exceptional longevity.

## Figures and Tables

**Table 1:** Baseline characteristics and follow-up of the study participants.

Variables	Men	Women	Total
Participants, n	100	100	200
Age at recruitment, years	92.8 ± 4.1	94.5 ± 4.6	93.6 ± 4.4
Age range at recruitment, years	90–106	90–106	90–106
Follow-up time, years	3.69 ± 1.81	2.85 ± 1.72	3.27 ± 1.81
Total follow-up, person–years	368.7	284.7	653.4
Crude mortality rate per 100 person-years	27.12	35.12	30.61
CIRS–derived comorbidity count ^1^	1.74 ± 1.08	1.86 ± 1.21	1.80 ± 1.14
MMSE ^2^ score	19.05 ± 6.61	19.81 ± 6.67	19.43 ± 6.63
BADL ^3^ score	4.02 ± 1.71	3.89 ± 1.54	3.96 ± 1.63
Calf circumference, cm	32.07 ± 4.66	31.96 ± 4.40	32.02 ± 4.52
Walking outside the home ≥3 times/week, n (%)	64 (64.0)	54 (54.0)	118 (59.0)

^1^ Cumulative Illness Rating Scale-derived comorbidity count based on the presence or absence of disease across 14 medical categories; disease severity was not graded; ^2^ MMSE, Mini-Mental State Examination; ^3^ BADL, Basic Activities of Daily Living.

**Table 2:** Varimax-rotated component loadings for the four dietary patterns identified by principal component analysis.

Food Item	PC1	PC2	PC3	PC4	Communality
Beef/pork meat	−0.190	**0.672**	0.100	−0.157	0.522
Sheep/goat meat	−0.018	**0.686**	−0.206	−0.231	0.566
Chicken meat	−0.067	−0.079	**0.780**	−0.072	0.625
Fish	−0.008	0.085	**−0.466**	−0.092	0.232
Pulses	−0.163	−0.131	0.375	**0.467**	0.403
Leafy greens	−0.115	**0.449**	−0.154	0.073	0.244
Fruit	−0.168	0.399	−0.154	**0.554**	0.519
Bread	0.328	0.350	**0.463**	0.371	0.583
Pasta	−0.087	−0.241	**0.481**	−0.202	0.338
Potatoes	−0.110	−0.240	−0.226	**0.679**	0.582
Olive oil	**−0.673**	0.096	0.201	−0.170	0.532
Lard	**0.436**	**−0.487**	0.147	0.043	0.451
Sweets	0.396	**−0.462**	**0.484**	−0.090	0.613
Cheese	**0.787**	−0.234	0.047	−0.118	0.690
Milk	**0.786**	−0.214	−0.099	−0.083	0.680
Coffee	**−0.465**	−0.082	−0.379	0.005	0.366
Wine	−0.243	0.096	−0.096	**−0.635**	0.481

Loadings with an absolute value ≥ 0.40 are shown in bold and were considered relevant for the interpretation and labeling of the components.

**Table 3:** Associations between continuous dietary-pattern scores and all-cause mortality estimated using Cox proportional hazards regression.

Dietary Pattern	Model 1, HR (95% CI) ^1^	Model 2, HR (95% CI) ^2^	Model 3, HR (95% CI) ^3^	*p*-Value ^4^	FDR *q*
Dairy–traditional fat	0.785 (0.684–0.901)	0.837 (0.725–0.966)	0.839 (0.727–0.969)	0.017	0.066
Red meat	1.010 (0.879–1.162)	1.048 (0.909–1.208)	1.043 (0.903–1.204)	0.566	0.928
Chicken–carbohydrate	0.868 (0.753–1.001)	0.988 (0.862–1.133)	0.984 (0.858–1.129)	0.821	0.928
Plant–starchy food	0.931 (0.799–1.084)	0.993 (0.855–1.154)	0.993 (0.855–1.154)	0.928	0.928

Hazard ratios are expressed per 1-SD increase in the continuous dietary-pattern score. ^1^ Model 1 was unadjusted. ^2^ Model 2 was adjusted for age at recruitment and sex. ^3^ Model 3 was adjusted for comorbidity burden measured using the CIRS-derived comorbidity count. ^4^ Nominal *p*-values and Benjamini–Hochberg false-discovery-rate-adjusted *q*-values refer to Model 3; correction was applied across the four dietary-pattern tests. All tests were two-sided. Abbreviations: CI, confidence interval; CIRS, Cumulative Illness Rating Scale; FDR, false discovery rate; HR, hazard ratio; SD, standard deviation.

## Data Availability

The original contributions presented in this study are included in the article/Supplementary Material. Further inquiries can be directed to the corresponding author.

## Supplementary Materials

The following supporting information can be downloaded at: https://www.mdpi.com/article/10.3390/nu18183072/s1, Figure S1: Scree plot and Horn’s parallel analysis; Figure S2: Principal component analysis loading plots of dietary patterns.; Table S1: Baseline characteristics of participants according to recruitment wave; Table S2: Pairwise comparisons of Kaplan–Meier survival distributions across tertiles of dietary-pattern scores; Table S3: Associations between tertiles of dietary-pattern scores and all-cause mortality estimated using Cox proportional hazards regression; Table S4: Sensitivity analysis of associations between continuous dietary-pattern scores and all-cause mortality after additional adjustment for cognitive status, functional autonomy, and calf circumference; Table S5: Sensitivity analyses of continuous dietary-pattern scores after exclusion of early deaths; Table S6: Sensitivity analyses of associations between continuous dietary-pattern scores and all-cause mortality according to physical activity and recruitment wave; Table S7: Associations between individual food-consumption frequencies and all-cause mortality estimated using Cox proportional hazards regression [35,36].

## Abbreviations

The following abbreviations are used in this manuscript:

CI	Confidence interval
CIRS	Cumulative Illness Rating Scale
FDR	False discovery rate
HR	Hazard ratio
IQR	Interquartile range
PCA	Principal Component Analysis
SD	Standard deviation
WHO	World Health Organization
