# Supplementary material for: Late-Life Dietary Patterns and Subsequent Survival Among Adults Aged 90 Years or Older in the Sardinian Blue Zone
Source: Nutrients. 2026 Sep 20;18(18):3072. doi: 10.3390/nu18183072 (PMC13610907; doi:10.3390/nu18183072)
Supplement: Supplementary file 1 [file nutrients-18-03072-s001.zip › Table S1.pdf]

Supplementary Table S1. Baseline characteristics of participants according to recruitment wave.

Variables	2018 wave, n = 150	2023 wave, n = 50	Absolute SMD
Age at recruitment, years	92.33 ± 3.33	97.48 ± 5.09	1.20
Men, n (%)	61 (40.7)	39 (78.0)	0.82
Age ≥ 100 years, n (%)	10 (6.7)	25 (50)	1.10
CIRS–derived comorbidity count	1.85 ± 1.22	1.66 ± 0.87	0.18
MMSE score	19.98 ± 6.02	17.78 ± 8.04	0.31
BADL score	3.99 ± 1.66	3.84 ± 1.54	0.10
Calf circumference, cm	31.91 ± 4.35	32.32 ± 5.04	0.09

Values are mean ± SD or n (%). SMD, standardized mean difference; CIRS, Cumulative Illness Rating Scale; MMSE, Mini-Mental State Examination; BADL, Basic Activities of Daily Living.
