# Supplementary material for: Late-Life Dietary Patterns and Subsequent Survival Among Adults Aged 90 Years or Older in the Sardinian Blue Zone
Source: Nutrients. 2026 Sep 20;18(18):3072. doi: 10.3390/nu18183072 (PMC13610907; doi:10.3390/nu18183072)
Supplement: Supplementary file 1 [file nutrients-18-03072-s001.zip › Table S2.pdf]

Supplementary Table S2. Pairwise comparisons of Kaplan–Meier survival distributions across tertiles of dietary-pattern scores.

**Dietary pattern**	**Comparison**	**Unadjusted p-value**	**BH-adjusted *q*-value**
Dairy–traditional–fat	T2 vs. T1	0.3511	0.3511
Dairy–traditional–fat	T3 vs. T1	0.0004	0.0013
Dairy–traditional–fat	T3 vs. T2	0.0086	0.0129
Red–meat	T2 vs. T1	0.5395	0.5395
Red–meat	T3 vs. T1	0.2696	0.4044
Red–meat	T3 vs. T2	0.1914	0.4044
Chicken–carbohydrate	T2 vs. T1	0.9821	0.9821
Chicken–carbohydrate	T3 vs. T1	0.1470	0.2205
Chicken–carbohydrate	T3 vs. T2	0.1119	0.2205
Plant–starchy–food	T2 vs. T1	0.8234	0.9788
Plant–starchy–food	T3 vs. T1	0.4509	0.9788
Plant–starchy–food	T3 vs. T2	0.9788	0.9788

[1] Pairwise comparisons were performed using log-rank tests. Within each dietary pattern, the three pairwise p-values were adjusted using the Benjamini–Hochberg procedure. T1, lowest tertile; T2, middle tertile; T3, highest tertile.
