# Supplementary material for: Late-Life Dietary Patterns and Subsequent Survival Among Adults Aged 90 Years or Older in the Sardinian Blue Zone
Source: Nutrients. 2026 Sep 20;18(18):3072. doi: 10.3390/nu18183072 (PMC13610907; doi:10.3390/nu18183072)
Supplement: Supplementary file 1 [file nutrients-18-03072-s001.zip › Table S3.pdf]

Supplementary Table S3. Associations between tertiles of dietary-pattern scores and all-cause mortality estimated using Cox proportional hazards regression.

Dietary pattern	HR [1] (95% CI)		HR (95% CI)	
	Tertile 2 vs tertile 1	P-value	Tertile 3 vs tertile 1	P-value
Dairy–traditional–fat	0.996 (0.698–1.420)	0.980	0.682 (0.480–0.969)	**0.033**
Red–meat	1.092 (0.763–1.562)	0.631	1.178 (0.826–1.682)	0.365
Chicken–carbohydrate	1.298 (0.907–1.857)	0.153	0.983 (0.691–1.399)	0.926
Plant–starchy–food	1.041 (0.709–1.527)	0.838	1.210 (0.878–1.667)	0.244

[1] Hazard ratios compare participants in the second and third tertiles with those in the first tertile, which served as the reference category. Models were adjusted for age at enrolment, sex, and comorbidity burden measured using the CIRS score. Global p-values were obtained using likelihood-ratio tests for the overall tertile variable. The four global tests were corrected using the Benjamini–Hochberg false-discovery-rate procedure. CI, confidence interval; CIRS, Cumulative Illness Rating Scale; FDR, false discovery rate; HR, hazard ratio.
