# Supplementary material for: Late-Life Dietary Patterns and Subsequent Survival Among Adults Aged 90 Years or Older in the Sardinian Blue Zone
Source: Nutrients. 2026 Sep 20;18(18):3072. doi: 10.3390/nu18183072 (PMC13610907; doi:10.3390/nu18183072)
Supplement: Supplementary file 1 [file nutrients-18-03072-s001.zip › Table S4.pdf]

Supplementary Table S4. Sensitivity analysis of associations between continuous dietary-pattern scores and all-cause mortality after additional adjustment for cognitive status, functional autonomy, and calf circumference.

Dietary pattern	Primary adjusted HR (95% CI) [1]	Extended-adjustment HR (95% CI) [2]	p [2]	FDR q [2]
Dairy–traditional-fat	0.839 (0.727–0.969)	0.852 (0.736–0.986)	0.032	0.127
Red-meat	1.043 (0.903–1.204)	1.067 (0.922–1.235)	0.367	0.735
Chicken–carbohydrate	0.984 (0.858–1.129)	1.024 (0.880–1.191)	0.638	0.851
Plant–starchy-food	0.993 (0.855–1.154)	1.004 (0.851–1.185)	0.958	0.958

Notes: Hazard ratios are expressed per 1-SD increase in the continuous dietary-pattern score. All analyses included 200 participants.

[1] Primary model adjusted for age at enrolment, sex, and the CIRS-derived comorbidity count.

[2] Extended model additionally adjusted for Mini-Mental State Examination (MMSE) score, Basic Activities of Daily Living (BADL) score, and calf circumference. FDR q-values were calculated using the Benjamini–Hochberg procedure across the four dietary-pattern tests in the extended model.

Abbreviations: CI, confidence interval; CIRS, Cumulative Illness Rating Scale; FDR, false discovery rate; HR, hazard ratio; SD, standard deviation.
