# Supplementary material for: Late-Life Dietary Patterns and Subsequent Survival Among Adults Aged 90 Years or Older in the Sardinian Blue Zone
Source: Nutrients. 2026 Sep 20;18(18):3072. doi: 10.3390/nu18183072 (PMC13610907; doi:10.3390/nu18183072)
Supplement: Supplementary file 1 [file nutrients-18-03072-s001.zip › Table S5.pdf]

Supplementary Table S5. Sensitivity analyses of continuous dietary-pattern scores after exclusion of early deaths.

Exclusion period	Pattern	N	HR [#] (95% CI)	p	FDR q
First 6 months	Dairy–traditional-fat	192	0.852 (0.736–0.985)	0.031	0.124
	Red-meat	192	1.067 (0.922–1.235)	0.384	0.767
	Chicken–carbohydrate	192	0.988 (0.858–1.137)	0.865	0.865
	Plant–starchy-food	192	0.984 (0.843–1.149)	0.841	0.865
First year	Dairy–traditional-fat	172	0.865 (0.742–1.008)	0.063	0.252
	Red-meat	172	1.099 (0.942–1.282)	0.232	0.463
	Chicken–carbohydrate	172	1.024 (0.880–1.191)	0.758	0.962
	Plant–starchy-food	172	1.004 (0.851–1.185)	0.962	0.962

[#] Hazard ratios are expressed per 1-SD increase in the continuous dietary-pattern score and are adjusted for age at recruitment, sex, and CIRS-derived comorbidity count. FDR q-values were calculated using the Benjamini–Hochberg procedure across the four dietary-pattern tests separately within each exclusion analysis.
