# Supplementary material for: Late-Life Dietary Patterns and Subsequent Survival Among Adults Aged 90 Years or Older in the Sardinian Blue Zone
Source: Nutrients. 2026 Sep 20;18(18):3072. doi: 10.3390/nu18183072 (PMC13610907; doi:10.3390/nu18183072)
Supplement: Supplementary file 1 [file nutrients-18-03072-s001.zip › Table S6.pdf]

Supplementary Table S6. Sensitivity analyses of associations between continuous dietary-pattern scores and all-cause mortality according to physical activity and recruitment wave.

Panel A. Sensitivity models

Dietary pattern	Model	N	HR (95% CI)	p-value	FDR q-value
Dairy–traditional-fat	Primary Model 3	200	0.839 (0.727–0.969)	0.017	0.066
Dairy–traditional-fat	Model 3 + recruitment wave	200	0.838 (0.726–0.967)	0.016	0.062
Dairy–traditional-fat	Model 3 + physical activity (MOV)	200	0.832 (0.720–0.961)	0.012	0.050
Dairy–traditional-fat	Model 3 + recruitment wave + physical activity (MOV)	200	0.832 (0.720–0.961)	0.012	0.049
Dairy–traditional-fat	Wave 1 only	150	0.882 (0.747–1.043)	0.142	0.283
Red-meat	Primary Model 3	200	1.043 (0.904–1.204)	0.565	0.929
Red-meat	Model 3 + recruitment wave	200	1.041 (0.902–1.202)	0.583	0.987
Red-meat	Model 3 + physical activity (MOV)	200	1.031 (0.890–1.193)	0.685	0.897
Red-meat	Model 3 + recruitment wave + physical activity (MOV)	200	1.031 (0.890–1.194)	0.684	0.901
Red-meat	Wave 1 only	150	1.167 (0.985–1.382)	0.074	0.283
Chicken–carbohydrate	Primary Model 3	200	0.984 (0.858–1.129)	0.821	0.929
Chicken–carbohydrate	Model 3 + recruitment wave	200	0.984 (0.858–1.130)	0.824	0.987
Chicken–carbohydrate	Model 3 + physical activity (MOV)	200	0.966 (0.839–1.113)	0.634	0.897
Chicken–carbohydrate	Model 3 + recruitment wave + physical activity (MOV)	200	0.966 (0.839–1.113)	0.633	0.901
Chicken–carbohydrate	Wave 1 only	150	0.959 (0.814–1.130)	0.618	0.825
Plant–starchy-food	Primary Model 3	200	0.993 (0.855–1.154)	0.929	0.929
Plant–starchy-food	Model 3 + recruitment wave	200	0.999 (0.857–1.164)	0.987	0.987
Plant–starchy-food	Model 3 + physical activity (MOV)	200	1.010 (0.870–1.172)	0.897	0.897
Plant–starchy-food	Model 3 + recruitment wave + physical activity (MOV)	200	1.010 (0.868–1.174)	0.901	0.901
Plant–starchy-food	Wave 1 only	150	1.019 (0.851–1.221)	0.837	0.837

Panel B. Recruitment-wave interaction analyses

Dietary pattern	Wave 1 HR (95% CI)	Wave 2 HR (95% CI)	Wave 2:Wave 1 ratio of HRs (95% CI)	Intercation p-value	FDR q-value
Dairy–traditional-fat	0.878 (0.745–1.035)	0.725 (0.544–0.966)	0.826 (0.595–1.146)	0.252	0.504
Red-meat	1.167 (0.987–1.381)	0.790 (0.605–1.032)	0.677 (0.494–0.928)	0.015	0.061
Chicken–carbohydrate	0.964 (0.821–1.133)	1.042 (0.797–1.362)	1.081 (0.790–1.479)	0.628	0.741
Plant–starchy-food	1.015 (0.848–1.214)	0.959 (0.719–1.278)	0.945 (0.674–1.324)	0.741	0.741

Notes: Hazard ratios are expressed per 1-SD increase in the continuous dietary-pattern score.
Primary Model 3 was adjusted for age at recruitment, sex, and the CIRS-derived comorbidity count.
Recruitment wave was coded as Wave 1 (2018, Pes, G.M. et al. Sociodemographic, Clinical and Functional Profile of Nonagenarians from Two Areas of Sardinia Characterized by Distinct Longevity Levels. Rejuvenation Res 2020, 23, 341-348) versus Wave 2 (2023, Marche, C. et al. Is a plant-based diet effective to maintain a good psycho-affective status in old age? Results of a survey of a long-lived population from Sardinia. Nutr Neurosci 2024, 27, 382-391).
Physical activity (MOV) was coded 1 for walking outside the home at least three times per week and 0 for walking outside the home less than three times per week.
The Wave 1-only analysis included the 150 participants recruited in 2018; dietary-pattern scores were those derived and standardized in the full 200-participant cohort to preserve comparability with the primary analysis.
In Panel B, wave-specific HRs and the Wave 2:Wave 1 ratio of HRs were derived from Cox models containing a dietary-pattern score × recruitment-wave interaction term and adjusted for age, sex, and CIRS-derived comorbidity count.
Benjamini–Hochberg false-discovery-rate correction was applied across the four dietary-pattern tests separately for each sensitivity model and across the four pattern-by-wave interaction tests.
All Cox models used Efron handling of tied event times. All participants had complete data for the variables included in these analyses.
