# Supplementary material for: Late-Life Dietary Patterns and Subsequent Survival Among Adults Aged 90 Years or Older in the Sardinian Blue Zone
Source: Nutrients. 2026 Sep 20;18(18):3072. doi: 10.3390/nu18183072 (PMC13610907; doi:10.3390/nu18183072)
Supplement: Supplementary file 1 [file nutrients-18-03072-s001.zip › Table S7.pdf]

Supplementary Table S7. Associations between individual food-consumption frequencies and all-cause mortality estimated using Cox proportional hazards regression.

Food item	HR per 1-SD increase (95% CI) #	p-value	BH-adjusted *q*-value	HR per additional estimated weekly occasion (95% CI)
Beef/pork meat	1.029 (0.895–1.184)	0.6848	0.9725	1.016 (0.942–1.096)
Sheep/goat meat	0.988 (0.859–1.137)	0.8697	0.9725	0.993 (0.912–1.081)
Chicken meat	0.811 (0.697–0.943)	0.0065	**0.0275**	0.897 (0.830–0.970)
Fish	0.981 (0.835–1.153)	0.8200	0.9725	0.983 (0.851–1.136)
Pulses	1.187 (0.985–1.373)	0.0513	0.0725	1.148 (0.991–1.291)
Leafy greens	1.190 (0.990–1.397)	0.0630	0.0935	1.144 (0.998–1.296)
Fruit	1.046 (0.892–1.226)	0.5811	0.9725	1.022 (0.945–1.107)
Bread	1.021 (0.877–1.189)	0.7876	0.9725	1.016 (0.905–1.141)
Pasta	0.979 (0.845–1.134)	0.7772	0.9725	0.983 (0.869–1.110)
Potatoes	0.949 (0.822–1.094)	0.4693	0.9725	0.967 (0.883–1.059)
Olive oil	0.995 (0.862–1.149)	0.9430	0.9725	0.997 (0.909–1.093)
Lard	0.976 (0.836–1.139)	0.7548	0.9725	0.984 (0.890–1.088)
Sweets	0.997 (0.872–1.140)	0.9697	0.9725	0.999 (0.944–1.057)
Cheese	0.775 (0.663–0.906)	0.0014	**0.0077**	0.897 (0.839–0.959)
Milk	0.735 (0.631–0.857)	<0.0001	**0.0014**	0.880 (0.826–0.938)
Coffee	1.002 (0.876–1.146)	0.9725	0.9725	1.001 (0.932–1.075)
Wine	1.336 (1.144–1.561)	0.0003	**0.0022**	1.143 (1.064–1.228)

Each food item was examined in a separate Cox proportional hazards model adjusted for age at recruitment, sex, and comorbidity burden measured using the CIRS score. Foods were not mutually adjusted for one another. Primary HRs are expressed per 1-SD increase in estimated weekly consumption frequency. Estimates per one additional estimated weekly consumption occasion are provided as a secondary representation of the same associations. Q-values were calculated by applying the Benjamini–Hochberg false-discovery-rate procedure across the 17 food-specific models. Bold values remained statistically significant after FDR correction. CI, confidence interval; CIRS, Cumulative Illness Rating Scale; FDR, false discovery rate; HR, hazard ratio; SD, standard deviation
